# Elements of Pathological Anatomy; Illustrated by Numerous Engravings

**Published:** 1840-11

**Authors:** 


ART. VI.—*Elements of Pathological Anatomy;* illustrated by numerous engravings, etc. etc. By SAMUEL D. GROSS, M. D. Boston: 1839.

---

(*Continued from the October No.*)

---

OUR present number will finish what we have to say on the first part of this comprehensive work.

The second section of the chapter on heterologous formations, is devoted to melanosis. In classing this with those productions, our author has conformed to the prevailing arrangement of pathological anatomists; but, we must confess, that we do not clearly see the propriety of such an assignment. In fact it is made in violation of the great division of morbid structures, into those which are like, and those which are unlike, any of the existing tissues; inasmuch as the melanotic substance seems most closely to resemble, if not indeed to be identical with the brown or black pigment met with in some parts of the body, as a normal element—for example, in the rete mucosum of the negro, and in the choroid. Its secretion and deposit where it should not be found is, at most, but an *error loci,* and cannot render it a heterologous production. Again—it can scarcely be denounced as malignant, as its history with a few exceptions, shows it to be destitute of the attributes which belong to that class of tumours. And these exceptions, it is probable, are but apparent, the melanotic secretion being, perhaps, associated with spongoid, carcinomatous or tuberculous action. The reference of the melanotic tumour to this or that class of morbid productions is not, however, a matter of much consequence; especially as no rule of practice is founded upon its classification.

In the six or eight pages which our author has devoted to melanosis, he has given to the student a lucid and comprehensive view of its natural, chemical and pathological history. It has been observed in several domestic animals. Professor Gross has seen it repeatedly in the ox, and others have met with it in the dog, cat, rabbit, rat, mouse, and some birds; but the horse has presented it more frequently than other animals, and in larger masses. White horses are oftener affected than those of any other color; a fact which seems to show, that the coloring matter of the skins of these animals, is developed in the blood, and not being deposited in its proper nidus is accumulated in masses in other parts.

In the human subject, it affects both sexes and all ages, but is far more frequent in the old than the young. In its chemical constitution, melanotic secretion bears much resemblance to black or venous blood; but offers in this respect considerable variety. In its physical characters, it also presents diversities of color and consistence; varying from brown to jet black, and from the consistence of ink to that of fibro-cartilage. When exposed to the action of the atmosphere it dries and becomes brittle. Now and then it is punctiform, or in dots disseminated over, or rather in a membrane ; and at other times it is lamellated ; but its more common form is that of globoid masses, which are sometimes solitary, and at others aggravated. These masses generally have an envelope of cellular or serous-like membrane, giving them an encysted appearance. In the opinion of Professor Gross, they are never organized. They cannot be injected, and if they occasionally show the rudiments of a fibrous structure, it must be regarded as cellular substance into which the melanotic matter has been effused.

"When the tumour is developed on the serous surfaces, it frequently presents a pedunculated appearance, like certain polypi of the uterus and the vagina, (Pl. II, Fig. 2.) In such cases it is always surrounded by a distinct cyst, of which it is difficult to say whether it be a new formation, or simply an extension of the natural membrane. There is another variety of melanotic tumour in which the covering seems to be formed by condensed fibrin, effused, in all probability, in consequence of the irritation excited by the presence of the foreign matter. Sometimes the cyst is of considerable thickness, firmly connected with the circumjacent tissues, and furnished with minute vessels ; generally, however, it is remarkably thin, soft, flocculent and without the least visible trace of organization. This variety of melanosis occurs most commonly in the liver and the brain. It is extremely rare. Carswell states that he never has seen an instance of it, and Laennec appears to have met with it only twice. I have noticed it several times in the liver of the ox."

The adipose and cellular tissues are most frequently the seats of this deposit. The organs in which it is oftenest found, are the lungs, ovaries and liver. Now and then it is so far a constitutional affection, as that it will appear simultaneously, or in rapid succession, in various parts of the body. This constitutes or indicates the melanotic diathesis of the systematic writers. Three opinions concerning the nature of this diathesis may be sustained. First, that the blood is in fault ; second, that the solids of the body generally have undergone a lesion of their vital properties, and the secretory function is thereby transformed; third, that a single organ has suffered such a lesion, and the others are affected sympathetically. Many facts are wanting to a satisfactory decision of these questions. Mean while we incline to the first opinion.

Of the remote causes which determine the production of melanosis we know very little. We have mentioned that the aged are most liable. A melancholic temperament favors its production. A sudden suppression of the catamenia has been followed by its development. Warm climates are said to present it oftener than cold. Finally, Dr. Hodgkin and Mr. Bransby Cooper have observed it in connexion with scorbutus.

"Melanotic tumors, after having acquired a certain size, generally remain stationary, giving rise to little or no inconvenience, save what results from their bulk and consequent pressure. At times, however, they manifest a disposition to ulcerate, and, when this happens, a most intractable sore is left, with hard, ragged edges, from the surface of which there is a constant discharge of black, inky matter, mixed with blood, pus, or a thin, fetid, ichorous fluid, formed by the surrounding structures. When removed, the most remarkable feature of these tumours is their tendency to re-appear in the neighborhood of the cicatrix, or in some remote organ."

In conclusion we may add that no remedy for melanosis, either topical or constitutional, has yet been discovered.

The third section of our author's chapter on heterologous formations, is devoted to scirrhus and cancer. We shall commence its analysis by transcribing his definition of the former, of which the latter is but a subsequent stage.

"How far the definition which we are about to give is free from objection, must be left to others to determine. Those who are acquainted with the difficulty of the subject will agree with me at least, that any effort of the kind, although it may only be approximately correct, is much better than none. No lesion can be studied with advantage, unless the student have a proper notion of its nature at the very outset of his examination; and nowhere is this more true than in the disease before us. With these remarks, I proceed to define scirrhus to be a hard, crisp, opaque substance, of a light grayish color, with dull yellowish fibrous intersections, organized, liable to lancinating pain, occurring for the most part after the middle period of life, and passing sooner or later into ulceration."

Scirrhous matter sometimes appears on surfaces in layers, at other times it is infiltrated into a tissue, converting the whole into a lardaceous or pork-like appearance, but more commonly it is deposited in tuberoïd masses, which may vary exceedingly in size, before softening and ulceration take place.

We were lately consulted in two cases of cancer in the face. One, which had nearly destroyed the under lip, was preceded, for a long time, by a small hard scirrhus, which felt like a shot beneath the epithelium of the lip. The other appeared as a circumscribed, scaly, and indurated spot, in the skin of one of the temples. At the time of our inspection, the ulcerated surface was several inches in diameter, and the tuberous granulations of such magnitude, as to present the characters of a spongoïd disease. This patient, an aged man, had some time before placed himself under the care of a *cancer doctress,* who attempted and predicted its cure; but failing to accomplish the object, she changed her diagnosis, and declared that *it could not be cancer or her application would have cured it.* We mention the incident as a diagnostic of that empiricism, which has so firm a hold on the confidence of the victims of this deplorable malady. But a few months before, we had seen a still more revolting, because a more advanced case of the same disease, in the Louisville Poor House, which, as the patient stated, began as a slight fissure of the lower lip. The mention of these cases reminds us, that all the examples of cancer of the lips and cheeks which we have ever seen, and the number has not been small, were in *men*, and that we have scarcely met with the disease in *women*, in any other parts than the breast and os tincæ. But to return from this digression, we shall extract from our author a paragraph on the most common of the different forms of scirrhus:

"In the tuberoïd variety, the most common, as we have already said, of all the heterologous substances, forms small circumscribed nodules, the number of which, as in the liver, is sometimes very great, and the consistence of which varies between fresh pork and fibro-cartilage. Their size and shape are much influenced by the nature of the tissues in which they are developed, and by the resistance which is offered to their progress. Single tumours of this kind are rounded, ovoïdal, or conical, when, on the contrary, several are agglomerated together, they are generally very irregular, angular, and more or less lobulated. In their size they vary from a mustard seed to an adult head,—their avarage volume being that of a billiard-ball, a lemon, or an orange."

Scirrhus exerts upon the skin a very different action from non-malignant encysted tumours, in becoming adherent to it, and at length giving it more or less of a puckered appearance. Previously to complete softening and ulceration, when examined, after being removed, scirrhous tumours sometimes contain hydatids, or coagula of blood, or cysts filled with gelatinoid or melicerous, or even purulent matter. In many cases, scirrhus presents an assemblage of lobulated masses, compacted together, but separated by processes of cellular or *quasi* serous membrane; the whole being surrounded by a capsule of the same kind, appearances which have led Dr. Hodgkin to the conclusion, that there is a close anatomical resemblance between this disease and some varieties of encysted dropsy. The conclusions of this able pathologist and benevolent man have not, however, been generally adopted by the profession. The lobulated arrangement to which we have referred presents a considerable variety, which has given rise to the epithets mammary, pancreatic, gelatinoid, &c. These varieties result partly from the stage of the disease, and partly from the texture of the organ affected.

"Scirrhus rarely appears before the age of thirty, in which respect it strikingly differs from encephaloïd. It is much more common in women than in men, and its favorite period for attack is from the fortieth to the fiftieth year. Rarely, perhaps *never*, does it occur before the period of puberty. The lymphatic temperament is said to predispose to it, and in some instances it seems to be connected with a hereditary taint, being transmitted from parent to offspring. In the uterus, mammary gland, and testicle, it has been repeatedly observed in three or four members of the same family. Very often it supervenes upon external violence, such as a blow, kick, or bruise, syphilitic disease, suppression of the menses, and the repulsion of herpetic eruptions. In other cases, again—and these are very common—it arises without any assignable cause. Corroding cares, by impairing the general health, sometimes induce this disease; and, in the female, it is often dependent upon sympathetic action between the uterus and the breast."

Scirrhus often attacks a considerable number of organs at the same time or progressively, and most commonly, it is said, selects those of a glandular structure. If, however, by glandular structure be meant *a secreting* structure, we are not entirely prepared to acquiesce in the conclusion. The salivary glands are seldom affected, the lungs and kidneys which respectively secrete more, perhaps, than any other organs of the body, are equally exempt. On the other hand, however, the mammæ and testes are exceedingly common seats of this complaint. Our own observations would lead us to think the liver is not often affected. We are almost disposed to say, that parts endowed with great sensibility and possessing much erectile tissue are peculiarly liable. This would at least be true of the lips, the stomach, the rectum, the cervix uteri, the mammæ and the testes; parts which are undoubtedly oftener the seats of scirrhus than all the other organs of the body.

Professor Gross, in contemplating the origin of scirrhus, inclines to the opinion of the distinguished Professor Carswell, that it begins with a deposit of lymph, which at length becomes organized, having two sets of vessels, one formed in the mass itself, the other derived from the surrounding arteries. We have already expressed our doubts of the validity of this theory; which, if adopted, explains only the origin of the primary nodule, and makes no provision for the *malignant* ulceration which follows on its softening; or for the lesion of the constitution, which constitutes the cancerous diathesis.

"After having existed for some time, varying from a few months to several years, the scirrhous matter manifests a disposition to become soft, the process by which this is effected, like that of tubercle, commencing at different parts of the diseased mass, from which it extends in various directions, until the whole or the greater portion of it is broken up and dissolved. Some authors have contended that the liquefaction invariably begins in the centre; but that this is not true, the writings of pathologists abundantly attest. The process, then, may commence at any point, at the centre or at the periphery, or simultaneously in both these situations; and, as it advances, the superincumbent integuments crack at one or more places, through which the softened matter, now of the aspect of encephaloïd, jelly, syrup, gum, or honey, is ultimately discharged. Ulceration, however, it should be observed, often occurs in scirrhous tumours long before the internal disorganization in question is accomplished.

"A scirrhous ulcer possesses certain features which may be considered as characteristic. Generally, it is remarkably irregular in its shape, with a surface that is either cracked, fissured, or fungous, of a dark reddish color, and of a peculiar glossy œdematous aspect. Soft cauliflower excresences sometimes sprout from it, so sensitive as to bleed on the slightest touch, or even of their own accord. The edges of the sore are of a reddish-gray color, elevated, everted, irregularly serrated, and harder in some places than in others, emitting more or less sanies on pressure. A deep excavation is occasionally formed, presenting the appearance as if a portion of the diseased mass had been lifted out of its bed. In cases which run their course very rapidly, the surface of the ulcer is frequently covered with a soft, grayish putrilage, of the most intolerable odor."

"In this advanced state of the disease, the skin around the ulcer is of a purple color—from the overloaded state of its capillaries—hard, puckered, somewhat tender on pressure, and easily corroded by the irritating discharges. By degrees, the ulcer spreads, both in depth and in diameter, until at length the whole mass is involved in the disorganizing process, and the patient sinks under the exhausting hectic, caused by the profuse local discharges, and by the violent constitutional irritation. The lymphatic ganglions, it should be further stated, in the neighborhood, are almost constantly enlarged and indurated, and the tumour, instead of being moveable and circumscribed, as it was in the early stage of its growth, forms a hard, solid, undefined mass, which firmly adheres to the surrounding structures."

We come now to the last of the heterologous products recognized by our author—ENCEPHALOÏD;—the well known Fungus Hæmatodes of Mr. Hey, by whose interesting paper, this affection was first made known to most of our physicians. Other synonymes are spongoïd inflammation, soft cancer, medullary fungus, and medullary sarcoma. Such a multiplicity of names is not a little perplexing to the student of pathology and surgery. Of the whole, we prefer that imposed by Mr. Hey.

According to our author, this morbid structure may present itself under three aspects, the stratiform, the infiltrated and the tuberous. The two former are rare—the last by no means uncommon, as every western practitioner can testify.

"In the tuberoïd variety, (Pl. IV, Fig. 1,) the heterologous matter appears in the form of a circumscribed tumour, from the size of a pea to that of a muskmelon. In its shape it is generally irregularly rounded, ovoïdal, or even quite flat, according to the amount of pressure that is exerted upon it by the surrounding parts; and, if it be examined by dissection, it will be found to be composed of different lobules, enveloped by a thin covering, and separated from each other by delicate membranous partitions. The outer covering, which is evidently formed out of the neighboring cellular tissue, is usually not more than half a line in thickness, easily torn, semi-transparent, and of a light rose color. From its inner surface are detached numerous processes, which, dipping into the morbid growth in various directions, form so many cavities for the reception of the new deposit. These septa, which are sometimes remarkably rough and shreddy, always become more obvious after the pulpy mass is squeezed out. The cells which they form by their intersections, are subject to much variety, and hence the peculiar lobulated shape which characterizes the morbid growth when occuring in parts that offer little or no obstacle to its extension.

"Although the covering of encephaloïd tumours is ordinarily derived from the pre-existing structures in their immediate vicinity, yet cases occasionally occur in which it is evidently of new formation. That this is true, my own dissections fully convince me. In such cases the external envelope is generally very thin; sometimes, indeed, almost like a film, easily lacerated, and of a grayish color, with rough, shreddy surfaces. The interior septa are likewise less perfect, and the whole mass is commonly so soft as to yield to the slighest force.

"The external envelope, together with the internal processes here described, is abundantly supplied with vessels which pervade the diseased mass in different directions, assist in its growth, and maintain its vitality. These vessels, which always consist of a much greater number of veins than of arteries, are often remarkably large and convoluted, and can be easily traced to the neighboring vascular trunks: their walls are exceedingly brittle, and the most trifling accident is therefore liable to be attended with an effusion of blood. Hence the dark clots which are so frequently met with in encephaloïd tumours. The cerebriform substance itself, it should be stated, is easily squeezed out of its cavities, owing to its imperfect adhesion; and, intespersed through different parts of it, we frequently observe, besides the sanguineous deposits just adverted to, small cells filled with purulent matter, serum, or thin, sanious, and offensive fluid. In one case, I saw as much as half a pint of reddish serosity flow from a single cavity, the inner surface of which had a peculiar honeycomb-like appearance."

In the opinion of Andral the term fungus hæmatodes is, in fact, generic, and has been applied by different anatomists to very different morbid productions. One of them is a hypertrophy of the erectile tissue. The following are his remarks on this subject.

"When these vessels are thus increased in size, and therefore apparently in number, they sometimes undergo a peculiar modification in their texture, which gives rise to the formation of a tissue, for which I cannot find a better standard of comparison, than the structure of the spleen. In fact, we observe, just as in the spleen, numerous areolæ filled with blood, communicating freely with each other, and with large veins, the parietes of which, perforated in all directions, are divided at their termination, or rather origin, into simple filaments, losing themselves in the areolar tissue. In these veins and areolæ, the blood neither circulates nor stagnates, and in this way its quantity is exceedingly variable, and thus produces rapid changes in the consistence, form, and size of the tumour. In such cases, the blood not unfrequently escapes, and thus gives rise to copious hæmorrhages.

"The blood which is found in the areolæ presents the same varieties in its appearance, consistence and color, as the blood effused into the cells of the spleen; thus, in different tumours, or parts of the same tumour, it appears colorless, pale-red, greyish, like the lees of wine, or as black as the pigment of the choroid; it is perfectly fluid, of the consistence of currant jelly, or hard as a piece of muscle; sometimes it is impossible to separate it from the solids which invest it, sometimes it is easily removed by washing and pressure, the parenchyma in which it was contained then presenting exactly the appearance of a spongy texture: in all these respects it perfectly resembles the appearance and structure of the splenic parenchyma.

"Such is the morbid alteration which has been described under the various appellations of fungus hæmatodes, sanguineous tumour, and more recently *accidental erectile tissue.* In the midst of this vascular development, other lesions of nutrition or secretion may likewise occur; hence it is that in several tumours of fungus hæmatodes, there have been found, in addition to the peculiar anastomosis of vessels constituting its basis, different morbid productions, such as fibrous or scirrhous masses, pus, melanosis, &c. Similar tumours are, not uncommonly, formed in the cutaneous tissue, and more frequently still in the subcutaneous and intermuscular cellular tissue. They are also found in the mucous membranes and their subjacent tissues. Of all the parenchymatous organs, the testicle appears most liable to this disease, which constitutes in it one of the varieties of sarcocele. I recollect having seen, in the Hospital La Charite, a number of erectile tumours developed in the lungs of an individual, who had had one of his testicles removed several months before, in consequence of its being the seat of a tumour that was likewise of an erectile nature. The tumours in the lungs, five or six in number, were each about the size of a walnut: they were embedded in the substance of the lung, which was perfectly healthy all around them; and in the points they occupied, it looked exactly as if the parenchyma of the spleen had taken the place of the pulmonary structure. In another case, for the particulars of which I am indebted to Professor Marjolin, a tumour, perfectly resembling a portion of the spleen, was found in the brain of an individual, who, like the patient at La Charite, had also had one of his testicles removed for the same affection, as was ascertained on examining it after the operation."

True fungus hæmatodes is generally a disease of early life. Our author has seen it occur soon after birth, and we have lately met with a case in which it was congenital. We have also seen it in a patient upwards of fifty years old. Professor G. has met with it oftener in females than males; such, however, has not been our own experience.

"The most common seats of this morbid growth are the bones, eyes, testicles, liver, lungs, kidneys, uterus, lymphatic ganglions, and subcutaneous cellular tissue. In infants it often occupies the shoulder, the region of the clavicle, the side of the chest, and the fore-arm. In adults I have seen a number of cases where it attacked the hand and fingers. Never have I observed it in the inferior extremities; but that it sometimes occurs here, the writings of pathologists abundantly testify. Encephaloïd has likewise been noticed in the veins, especially in those of the liver, of the kidney, and the uterus."

We have seen this malady attack the knee, the upper and inner part of the thigh, the soft parts about the ischium, the super-costal cellular substance between the axilla and the spine of the ilium, but above all the scapulary region which has seemed to us to be its favorite seat. But why this region should be more obnoxious than other cellular parts, we cannot tell. Since beginning this article, we have been consulted by a man whose case may be thus stated. He is thirty-one years of age, and by trade a cabinet-maker. About four years ago he discovered a small tumour in the right side, over the eleventh and twelfth ribs, which grew slowly for three years. Under exercise he felt some pain from the beginning, and tenderness on pressure. For the last year, he thinks it has rather decreased in size; but this is probably the result of the absorption of the healthy tissues in and around it. At this time it has an oval base, is but little elevated, feels knotted and corded within, and has formed attachments for the skin above, which is somewhat livid and marbled in its appearance, and exhibits many small varicose veins. From this tumour up to the shoulder there is a degree of fulness, which is quite perceptible to the eye when the patient is seen from behind. Indeed the scapula seems thrown outwards, and the sterno-spinal circuit of that side is an inch longer than the other. All round the margin of the scapula the parts are tender to the touch, and between that bone and the spine the pain has been such as to cause him to apply an adhesive plaster. This pain is increased by the motion of the arm. The axillary ganglia are unaffected. We take this to be an incipient fungus hæmatodes.

We have met with but one case of this disease affecting the mamma, and then it had extended to that organ from the axilla and shoulder. We have twice been tempted to extirpate the eyeball disorganized by this malady. In the first instance, that of a child, it was associated with such a degree of ossification as rather to interfere with the progress of the knife. A fungus shot up rapidly from the socket, and the disease went to a fatal termination. In the other case, that of a woman near fifty years old, there were strong symptoms of reproduction, when she passed from under our care to a distant state, whence we have not heard the final issue. When lately at Zanesville, we were shewn by our friend, Dr. Rhodes, a testicle affected with this malady which he had extracted and the patient afterwards remained exempt.

The following is our author's account of the progress of this malady:

Encephaloïd disease, after having attained a certain development, may remain stationary for years, unaccompanied by the slightest uneasiness, until the part receive some injury, when it often grows with frightful rapidity. When seated in the subcutaneous cellular tissue, the tumour that is thus formed is at first quite movable, smooth on the surface, and devoid of sensation; but gradually, as the enlargement progresses, it becomes stationary, irregularly lobulated, elastic to the touch, and more or less painful. If allowed to proceed, the diseased mass has a tendency to open and protrude, generally by ulceration, sometimes by sloughing, and occasionally by the bursting of an abcess situated in its interior. In either case, the exposed surface presents a dark reddish fungous appearance, is highly sensitive, extremely vascular, very prone to hemorrhage, and constantly bathed with a thin, fetid, irritating, sanious fluid, the quantity of which is sometimes quite profuse. In many instances, pure blood is effused, caused by a rupture of some of the vessels of the morbid growth; and this may be so obstinate and copious as gradually to destroy the patient. Occasionally there is a discharge of thin, glairy fluid, resembling the white of an egg. Such sores, besides being always highly disagreeable, never heal, from the inability of the parts to form healthy granulations. Sometimes the ulcerated mass sloughs as completely away as if it were dissected out; but these cases are uncommon, and are soon followed by a reproduction of the heterologous substance.

"Obstinate hemorrhage is most apt to occur in such tumours as are of the class to which Mr. Key applied the term fungus hæmatodes. In the eye, for example, much more frequently than elsewhere, the morbid growth, if it be permitted to go on unrestrained, is extremely prone to bleed. The reason of this is obvious. The diseased mass is almost always composed, in part, of a vascular, erectile tissue, interspersed with encephaloïd matter, and, as soon as ulceration sets in, hemorrhage, occasionally to an alarming and even fatal extent, is the consequence. The eroded surface, in these cases, is pale, livid, or mahogany color, and studded with large fungous excrescences, so grouped together as to resemble a cauliflower.

"In this advanced state of the disease, there is a rapid failure of the strength, the flesh wastes, the appetite declines, the patient is harassed with hectic fever, and the countenance assumes a peculiar yellowish, cadaverous hue. The lymphatic ganglions in the neighborhood meanwhile become enlarged, and converted into a substance resembling that of the original tumour. Two modes of explanation may be offered to account for this phenomenon. The one supposes that these bodies are effected merely sympathetically, in consequence of which their vessels pour out encephaloïd matter; the other, that this substance is carried to them by absorbing vessels coming from the affected part. Although this enlargement of the lymphatic ganglions seldom occurs before ulceration sets in, yet, in a few instances, I have known it to exist at an early period after the development of the heteroclite mass, a good while before the skin covering it manifested a disposition to give way."

We here close our analysis of the first part of Prof. Gross's Elements—that which treats of General Pathological Anatomy. The analysis of the second part will be commenced with our third volume. In thus continuing it through many successive numbers, our object is not so much to make known the merits of this very excellent treatise, as to make it the occasion of a series of papers, designed to promote the study of pathology and pathological anatomy, in a region of country where these branches, particularly the latter, have not hitherto received the attention they deserve. For this purpose it seems to us peculiarly proper to employ as our text, a work written among those for whom we purvey, especially when we are compelled to regard it as superior to any other within their reach.

If we are not greatly misinformed the *alumni* of our Western Schools, and justice requires us to add of our Eastern likewise, have been seldom examined on morbid structure. A knowledge of healthy anatomy and of symptomatology has been demanded, but who can attempt to justify the omission of pathological anatomy? We trust that the day is not distant when the *onus* of our examinations will rest on that branch; and that a diploma will not be granted to one who is not as well acquainted with the morbid as the healthy structure of the body. D.

